# Nicotine Delivery and User Ratings of IQOS Heated Tobacco System Compared With Cigarettes, Juul, and Refillable E-Cigarettes

**DOI:** 10.1093/ntr/ntab094

**Published:** 2021-05-13

**Authors:** Anna Phillips-Waller, Dunja Przulj, Francesca Pesola, Katie Myers Smith, Peter Hajek

**Affiliations:** Health and Lifestyle Research Unit, Queen Mary University of London, London, UK

## Abstract

**Introduction:**

Reduced-risk nicotine products are more likely to replace smoking if they match cigarettes in nicotine delivery and user satisfaction.

**Aims and Methods:**

We examined the nicotine delivery profile and user ratings of IQOS heated tobacco system and compared it with own brand cigarettes (OBC), Juul, and refillable e-cigarettes (EC).Participants (*N* = 22) who were daily vapers smoking <1 cigarette per day on average, attended after overnight abstinence from smoking and vaping, to test at separate sessions OBC, IQOS, and Juul. Eight participants also tested two refillable EC using e-liquid with 20 mg/mL nicotine. At each session, a baseline blood sample was taken before participants used the product ad libitum for 5 minutes. Further samples were taken at 2, 4, 6, 8, 10, and 30 minutes. Maximum nicotine concentration (*C*_max_), time to *C*_max_ (*T*_max_), and nicotine delivered over 30 minutes (AUC_0–>30_) were calculated. Participants rated their urge to smoke and product characteristics.

**Results:**

IQOS delivered less nicotine than OBC (AUC_0–>30_: *z* = −2.73, *p* = .006), and than Juul (AUC_0–>30_: *z* = −3.08, *p* = .002; *C*_max_: *z* = −2.65, *p* = .008), and received less favorable ratings than Juul (effect on urges to smoke: *z* = −3.23, *p* = .001; speed of urge relief: *z* = −2.75, *p* = .006; recommendation to friends: *z* = −2.45, *p* = .014). Compared with refillable EC, IQOS delivered nicotine faster (*T*_max_: *z* = −2.37, *p* = .018), but received less favorable overall ratings (recommended to friends: *z* = −2.32, *p* = .021).

**Conclusions:**

IQOS’ pharmacokinetic profile suggests that it may be less effective than Juul for smoking cessation, but at least as effective as refillable EC; although participants, who were experienced vapers rather than IQOS users, preferred refillable EC.

**Implications:**

Because IQOS provided less efficient nicotine delivery than cigarettes and Juul in this sample, and also had a weaker effect on urges to smoke than Juul, it could be less helpful than Juul in assisting such dual users, and possibly smokers generally, to switch to an alternative product. IQOS, however, provided nicotine faster than refillable EC products, although participants preferred EC.

## Introduction

Heated tobacco products (“heat-not-burn,” HnB) aerosolize tobacco chemicals, including nicotine, by heating tobacco, rather than burning it. The process substantially reduces the release of harmful chemicals that make smoking hazardous, although it does not remove them altogether.^1^

The most popular HnB product is currently IQOS, first released by Philip Morris International (PMI) in Japan in 2014, and since then marketed in other countries as well.^2^ Japan saw a rapid increase in the popularity of IQOS, accompanied by a decrease in cigarette sales.^3^ IQOS is less popular in Europe, possibly because smokers in Europe have access to e-cigarettes (EC), while nicotine-containing EC are banned in Japan. IQOS was initially not authorized for sale in the United States, but in July 2020, the product obtained a licence to be marketed as a modified risk tobacco product.^4^

Reduced-risk nicotine delivery products have a potential to replace smoking, and thus reduce smoking-related morbidity.^5^ One of the key determinants of their success in this regard is whether they can deliver nicotine in the way that cigarettes do. A number of studies have compared nicotine content in aerosol from IQOS and from cigarettes.^6–13^ IQOS was reported to release 65%–96% of nicotine content that cigarettes release in its emissions, depending on the cigarette comparator and the setting of puffing regimes. Regarding nicotine delivery to users, a PMI study reported levels similar to those obtained from conventional cigarettes, when participants took 14 puffs over 6 minutes to completely use the full IQOS cartridge. Details of the instructions for using cigarettes and time of day when the experiment was conducted were not provided.^14^ An independent study that did not collect pharmacokinetic (PK) data, but measured blood nicotine levels before and after use, reported that in participants with no previous experience of alternative nicotine delivery products, IQOS delivered less nicotine than cigarettes after a scheduled 10-puff regimen, followed by ad libitum use.^15^ The contradictory results could be due to differences in puffing schedules, participants, or study timing. Regarding longer-term studies, a meta-analysis of 10 trials reported a significantly lower levels of nicotine in HnB users compared with smokers.^16^

We have earlier established a panel of participants who have tested a range of alternative nicotine delivery products, as well as cigarettes.^17–19^ The project is using a paradigm that, rather than using prescribed puffing, mimics “real-life” use, where products are used ad libitum over 5 minutes after overnight abstinence. In the present study, we tested the PK profile of nicotine delivery from, and user ratings of, IQOS, and compared IQOS with own brand cigarettes (OBC) and with other alternative nicotine delivery products including Juul (59 mg/mL nicotine), a product with high nicotine delivery, and traditional EC products.

## Methods

### Design

Within subjects, crossover laboratory study.

### Participants

Twenty-two vapers who also smoked at least occasionally were recruited through social media and word of mouth. Participants were eligible if they were willing to test a series of alternative nicotine delivery products, were willing to give blood samples, and had no serious illnesses.

### Procedures

Participants attended each session after overnight abstinence from both smoking and vaping (between 7.30 and 9.30 am, depending on their preference). Abstinence from cigarettes was validated with a carbon monoxide reading of less than 10 parts per million (ppm). The products were tested in the same order: OBC (which participants brought with them) was tested at the first session. Eight of the participants also tested two refillable EC products in an earlier study.^17,18^ This was followed by IQOS, and then Juul. Each session tested one product only, with at least 1 week wash out period between sessions.

At all sessions, an intravenous line was inserted into the participant’s forearm for blood sampling. A baseline blood sample was taken, after which participants were asked to smoke/vape as much or as little as they wanted for 5 minutes. Further blood samples were taken at 2, 4, 6, 8, 10, and 30 minutes after starting the product use. The sessions lasted on average 45 minutes. Participants received £60 per session for their time and travel.

Blood samples were centrifuged, frozen, and stored at the Health and Lifestyle Research Unit, QMUL for up to 7 days before being transported to ABS Laboratories Ltd, BioPark (Welwyn Garden City, UK) for analysis.

### Measures

Demographic and smoking/vaping data were collected at the baseline session. The number of puffs taken with each product was recorded. The following PK parameters were established for each product: maximum nicotine concentration (*C*_max_), time to maximum nicotine concentration (*T*_max_), and area under the curve, which measures the amount of nicotine delivered over 30 minutes (AUC_0–>30_). For completeness, we also report nicotine levels at baseline and nicotine boost effect (*C*_max_—baseline level) for the three products (see Supplementary Tables A and B).

Baseline urge to smoke was rated prior to product use on a scale of 1–10 (1 = no urge and 10 = extreme urge). Further ratings were collected 5, 10, 15, and 30 minutes after starting product use.

At the end of each session, product ratings were collected. At all sessions the following were asked: “Did the product relieve your urge to smoke?” [1 = not at all, 10 = extremely well]; “How quickly did any effect happen?” [1 = very slowly, 10 = extremely fast]; “How much nicotine do you think it delivered?” [1 = too little, 5 = just right, 10 = too much); “Did you like the taste?” [1 = not at all, 10 = extremely]; “Was it pleasant to use?” [1 = not at all, 10 = extremely]; and “How likely would you be to recommend it to friends?” [1 = not at all, 10 = extremely].

### Study Products

IQOS heated tobacco system (PMI) was tested with tobacco flavor HEETS sticks. The US version of Juul (59 mg/mL nicotine) was tested with Virginia Tobacco flavor (Juul Labs). Eight participants also tested two refillable EC: KangerTech EVOD and Innokin iTaste MVP 2 (variable voltage), set to 4.8 V (range = 3.3–5.0 V). The refillable products were tested with the same 20 mg/mL tobacco flavored e-liquid.

### Statistical Analysis

PK Solver add-in for Excel version 2.0^20^ was used to determine *C*_max_, *T*_max_, and AUC_0≥30_, using a noncompartmental analysis and trapezoidal rule.^21^ The 0-, 2-, 4-, 6-, 8-, 10-, and 30-minute blood samples were corrected for baseline nicotine levels. IQOS was compared with OBC and with Juul on the full sample using pairwise comparisons with Bonferroni adjustment (adjusted alpha = 0.05/2 = 0.025). The comparison of IQOS with refillable EC was tested separately on the reduced sample. The assumption that the difference between scores was normally distributed was assessed using the Shapiro–Wilk test, plus visual inspection of probability plots. As violation of this assumption may lead to biased results, the Wilcoxon signed rank test was used to test differences between the products’ scores. The effect size was estimated as r=z/sqrt(N).

To explore the effects of the products on urges to smoke, we used a linear mixed-effect model using the mixed command in Stata 16.1. Participants were treated as the cluster and random slopes and intercepts were used to account for repeated measures. We regressed follow-up urge scores onto product, time, and product × time interaction while adjusting for baseline urge scores. To compare the relevant products (eg, IQOS vs. Juul and IQOS vs. cigarettes) on urge scores, we estimated ANOVA-style test comparisons for product. To achieve this we used the contrast postestimation command in Stata, which reports these comparisons tests as chi-square tests.

Participants were treated as the cluster and random slopes and intercepts were used to account for repeated measures. We assessed IQOS versus Juul and IQOS versus cigarette comparisons while adjusting the alpha level using the Bonferroni approach (adjusted alpha = 0.05/2 = 0.025). For the mixed-effect model, the assumption of homoscedasticity was visually assessed by plotting the standardized residuals against the fitted values.

The study was not preregistered on a publicly available platform, so the results should be considered exploratory.

All analyses were carried out using SPSS version 25, except for the mixed-effect regression, which used Stata version 16.

The project was approved by the QMUL Ethics of Research Committee on April 3, 2018 (QMERC2018/09).

## Results

The participants were on average 31 years old, 82% (*N* = 18) were men, they smoked on average 14 cigarettes a day before becoming dual users but were now smoking less than one cigarette per day on average. They all vaped daily, with 19 (86%) using refillable devices, and have been using EC for 1 year on average at the start of the study (see Supplementary Table C).

**Figure 1.**
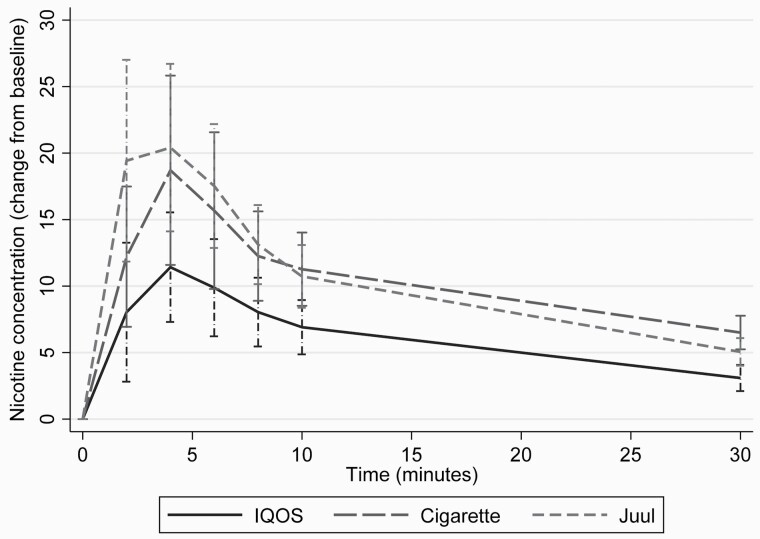
Pharmacokinetic profiles of IQOS, own brand cigarette and Juul (*N* = 22).

Figure 1 shows the nicotine delivery from IQOS, OBC, and Juul. Supplementary Figure A shows the urges to smoke after using the three products.

### Comparison of IQOS and OBC

IQOS delivered about half as much nicotine over 30 minutes (AUC_0–>30_) as a cigarette, over a similar number of puffs (see Figure 1 and Table 1).

**Table 1.** Nicotine Delivery and Number of Puffs Taken From IQOS, Own Brand Cigarette, and Juul (*N* = 22)

Product	Median no. of puffs (IQR)	Median *C*_max_ (IQR)	Median *T*_max_^a^ (IQR)	Median AUC_0–>30_^a^ (IQR), *N* = 21^b^
IQOS	14.0 (13.5–14.0)	8.3 (4.5–19.3)	4.0 (4.0–6.0)	152.0 (91.2–254.5)
Cigarette	13.0 (10.8–16.3)	12.9 (7.2–28.6)	6.0 (4.0–8.0)	314.7 (136.4–465.6)
Juul	13.0 (10.0–19.5)	19.6 (8.9–36.3)	4.0 (2.0–6.0)	343.2 (168.1–461.1)
Wilcoxon test, effect size				
IQOS vs. cigarettes	*z* = −0.10 *p* = .917 *r* = 0.02	*z* = −1.67 *p* = .095 *r* = 0.36	*z* = −2.16 *p* = .031 *r* = 0.47	*z* = −2.73 *p* = .006 *r* = 0.60
IQOS vs. Juul	*z* = −1.12 *p* = .265 *r* = 0.24	*z* = −2.65 *p* = .008 *r* = 0.56	*z* = −0.67 *p* = .505 *r* = 0.14	*z* = −3.08 *p* = .002 *r* = 0.67

IQR = interquartile range.

^a^Median *T*_max_ and AUC_0–>30_ values that were used to compare products differ slightly from values in Figure 1, because the comparisons here use medians across individuals, whereas PK Solver calculates means across timepoints.

^b^AUC_0–>30_ could not be calculated for one participant as the final blood sample could not be collected.

Three participants had baseline nicotine levels of over 10 ng/mL on at least one session, indicating they used a nicotine product late at night or in the morning. With these participants excluded in sensitivity analyses, the difference in AUC_0–>30_ between IQOS and OBC was no longer significant (*z* = −2.20, *p* = .028).

OBC reduced urges to smoke more than IQOS (see Supplementary Figure A, Chi2(1) = 5.1, *p* = .02).

### Comparison of IQOS and Juul

IQOS had a lower *C*_max_ and AUC_0–>30_ than Juul, over a similar number of puffs (see Table 1).

Excluding three participants who had baseline nicotine levels of over 10 ng/mL on at least one session did not change the results.

Regarding effects on urges to smoke (see Supplementary Figure A), the difference between IQOS and Juul was Chi2(1) = 3.5, *p* = .06.

Table 2 shows participant ratings of IQOS and Juul. IQOS was rated less favorably than Juul in terms of urge relief, speed of urge relief, and whether participants would recommend the product to friends.

**Table 2.** Participant Median Ratings (IQR) of IQOS and Juul (*N* = 22)

Product characteristic	IQOS	Juul	Wilcoxon test, effect size
Did it relieve your urge to smoke (1 = not at all, 10 = extremely well), median (IQR)	6.5 (6.0–9.0)	9.0 (8.0–10.0)	*z* = −3.23 *r* = 0.69 *p* = .001
How quickly did any effect happen? (1 = very slowly, 10 = extremely fast), median (IQR)	7.0 (4.8–8.0)	8.0 (7.0–9.3)	*z* = −2.75 *r* = 0.59 *p* = .006
How much nicotine do you think it delivered? (1 = too little, 5 = just right, 10 = too much), median (IQR)	5.0 (4.0–6.0)	5.5 (5.0–7.0)	*z* = −1.90 *r* = 0.40 *p* = .058
Did you like the taste? (1 = not at all, 10 =extremely), median (IQR)	5.5 (3.0–8.0)	7.0 (3.8–7.3)	*z* = −0.227 *r* = 0.05 *p* = .820
Was it pleasant to use? (1 = not at all, 10 = extremely), median (IQR)	6.5 (4.8–9.0)	7.0 (6.0–10.0)	*z* = −1.046 *r* = 0.22 *p* = .295
How likely would you be to recommend it to friends? (1 = not at all, 10 = extremely), median (IQR)	5.0 (2.0–7.0)	7.0 (6.0–9.3)	*z* = −2.453 *r* = 0.52 *p* =0.014

IQR = interquartile range.

### Comparison of IQOS With Refillable EC Products

**Figure 2.**
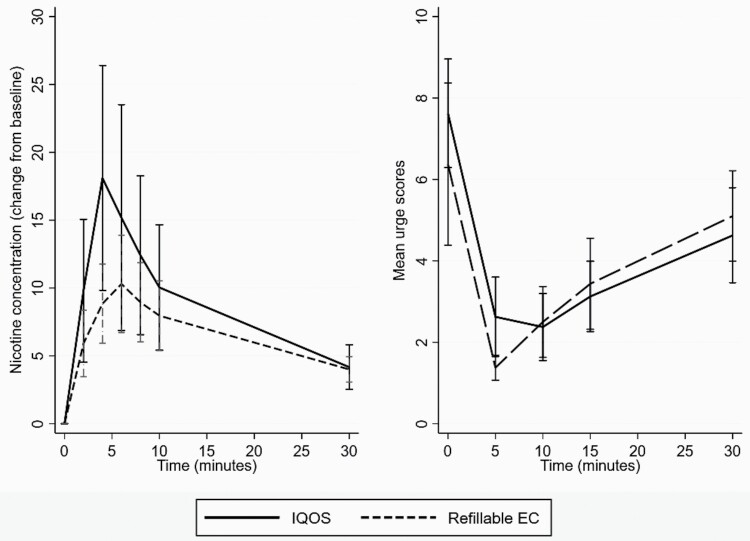
Pharmacokinetic profiles, and urges to smoke after using IQOS and refillable e-cigarette (*N* = 8).

Figure 2 shows the PK profiles of IQOS and the refillable EC which were tested earlier by eight of the participants, and the effects of the products on urges to smoke. As the two refillable EC had similar PK characteristics,^17^ their scores were averaged.

IQOS reached maximum nicotine concentration faster than refillable EC (see Supplementary Table D), but the products did not differ in effects on urges to smoke (Chi2(1) = 0.01, *p* = .91).

At the IQOS testing session, one participant had a baseline nicotine level of over 10 ng/mL, indicating they had used a nicotine product late at night or in the morning. Excluding this participant did not change the results.

Supplementary Table E shows ratings of IQOS and refillable EC. Participants were less likely to recommend IQOS to friends than refillable EC.

## Discussion

When used ad libitum for 5 minutes after overnight abstinence, IQOS heated tobacco system delivered less nicotine than OBCs. When compared with EC products, IQOS provided less nicotine than Juul and received less favorable user ratings, including effects on urges to smoke. IQOS however delivered nicotine faster than standard refillable EC using e-liquid with 20 mg/mL nicotine.

The nicotine delivery profiles on their own suggest that IQOS could be less effective in helping smokers quit than Juul, but at least on par with standard refillable EC products.

The efficacy for smoking cessation, however, may not rest exclusively on the degree to which a product’s nicotine delivery matches that of cigarettes. Other product characteristics, such as flavor and ease of use, are likely to also play a role.

IQOS did not differ from EC products in ratings of taste and pleasantness, but participants were less likely to recommend it to friends than both Juul and refillable EC. It could be argued that this was because the sample comprised of regular EC users, for whom IQOS was a less familiar product. It is possible that for smokers trying an alternative product for the first time, ie, not yet habituated to EC, the greater similarity of IQOS emissions to cigarette smoke could be more appealing than using an EC. However, the fact that IQOS is much more popular in Japan, where EC are banned, than it is in the EU, where EC are available, suggests that, overall, EC may have a stronger appeal to smokers than IQOS. This could be due to a greater versatility of EC in terms of tailoring nicotine strength, product flavors, and time patterns of use to smokers’ needs, or other product characteristics, such as ease of use and the smell of used IQOS cartridges. In countries where EC are more strictly regulated, eg, where EC flavors are banned, IQOS may become a more attractive choice.

The differences between our results and those from the two previous studies could be due to differences in puffing schedules, study timing, and especially participants’ experience with the products tested. The first cigarette of the day is smoked differently from later cigarettes.^22^ Dual users habituated to EC devices may use them differently from novice users.^23–26^ Sensory and conditioned effects of products new to users may mask nicotine effects, or lack of them, initially, with central nicotine effects becoming more important in determining user reactions later on.^18,27^ These considerations may also limit the generalizability of our results.

The study has several limitations. The sample comprised of EC users with no previous experience with IQOS, which could have affected their product ratings. The sample available for the comparison between IQOS and refillable EC products was relatively small, although not unusually so for this type of study. Products were tested in the same order, and so some influence of an order effect cannot be ruled out.

In conclusion, when used ad libitum after overnight abstinence, IQOS delivers less nicotine than cigarettes, and also less than Juul. It delivers nicotine at least as well as refillable EC products, but it may be less attractive to smokers, especially to those who have experience with EC use.

## Supplementary Material

A Contributorship Form detailing each author’s specific involvement with this content, as well as any supplementary data, are available online at https://academic.oup.com/ntr.

ntab094_suppl_Supplementary_Materials

ntab094_suppl_Supplementary_Taxonomy_Form

## Funding

The study was funded by a Tobacco Advisory Group project grant, Cancer Research UK (ref: C49913/A25756). DP, PH, and AP-W were in receipt of the funding.

## Declaration of Interests

*PH has received research funding from and provided consultancy to* Pfizer*, a manufacturer of stop-smoking medications. DP has received research funding from* Pfizer*. Other authors have no conflicts to declare.*

## Data Availability

Data are available upon request.
