# Supplementary material for: Nicotine Delivery and User Ratings of IQOS Heated Tobacco System Compared With Cigarettes, Juul, and Refillable E-Cigarettes
Source: Nicotine Tob Res. 2021 May 13;23(11):1889–94. doi: 10.1093/ntr/ntab094 (PMC8496472; doi:10.1093/ntr/ntab094)
Supplement: ntab094_suppl_Supplementary_Materials [file ntab094_suppl_supplementary_materials.docx]

**Supplementary Materials**

**Table A. Baseline nicotine levels**

|  | **Juul** | **IQOS** | **Cigarettes** |
| --- | --- | --- | --- |
| Baseline nicotine median (IQR) | 1.13 (0- 3) | 1.2 (0- 6) | 0.6 (0- 2.1) |
| Range (min- max) | 1. 21.5 | 1. 7 | 1. 22.5 |

**Table B. Nicotine boost effect (C_max_- baseline)**

|  | **Juul** | **IQOS** | **Cigarettes** |
| --- | --- | --- | --- |
| Boost score (C_max_-baseline) median (IQR) | 14.5 (7.2- 30.2) | 5.4 (2.6- 10.8) | 12.7 (6.7- 26.8) |

**Table C: Participant characteristics (N=22)**

| Male, N (%) | 18 (81.8) |
| --- | --- |
| Age, median (IQR) | 31.0 (27.8-46.0) |
| Higher education, N (%) | 13 (59.1) |
| Cigarettes smoked per day at start of study, median (IQR) | 0.7 (0.6-2.0) |
| Cigarettes smoked per day before starting vaping, mean (SD) | 13.9 (7.7) |
| Fagerstrom Test for Cigarette Dependence (FTCD) before starting vaping, mean (SD) | 4.0 (2.6) |
| Nicotine content of e-liquid (mg/ml) used at start of study, median (IQR) | 12.0 (6.0-17.3) |
| Amount of e-liquid used per day in mL at start of study (N=21), median (IQR) | 2.0 (1.2-4.2) |
| Number of months using EC daily at start of study, median (IQR) | 12.0 (2.0-36.0) |
| Number of days used EC in last week at start of study, median (IQR) | 1. (7.0-7.0) |

**Table D: Nicotine delivery and number of puffs taken from IQOS and refillable EC (N=8)**

| **Product** | **IQOS** | **Refillable EC** | **Wilcoxon test, effect size** |
| --- | --- | --- | --- |
| **Median no. of puffs (IQR)** | 14.0  (12.5-14.0) | 14.3  (10.9-17.9) | z=-0.85  r=0.30  p=0.397 |
| **Median C_max_ (IQR)** | 19.4  (6.4-28.5) | 12.6  (7.1-13.9) | z=-1.68  r=0.59  p=0.093 |
| **Median T_max*_ (IQR)** | 4.0  (4.0-5.5) | 7.5  (5.3-14.1) | z=-2.37  r=0.84  p=0.018 |
| **Median AUC_0->30*_ (IQR), N=7**** | 257.4  (145.7-390.1) | 199.3  (114.1-263.1) | z=-1.35  r=0.51  p=0.176 |

**Median T_max_ and AUC_0->30_ values that were used to compare products differ slightly from values in Figure 2, because the comparisons here use medians across individuals, whereas PK Solver calculates means across time-points.*

*** AUC_0->30_ could not be calculated for one participant as final blood sample could not be collected.*

**Table E: Participant ratings of IQOS and refillable EC (N=8)**

| **Product characteristic** | **IQOS** | **Refillable EC** | **Wilcoxon test, effect size** |
| --- | --- | --- | --- |
| Did it relieve your urge to smoke (1=not at all, 10=extremely well), median (IQR) | 9.0  (6.3-10.0) | 9.5  (7.8-10.0) | z=-1.27  r=0.45  p=0.206 |
| How quickly did any effect happen? (1=very slowly, 10=extremely fast), median (IQR) | 7.5  (6.3-8.0) | 8.3  (7.1-9.0) | z=-1.79  r=0.63  p=0.073 |
| How much nicotine do you think it delivered?(1=too little, 5=just right, 10=too much), median (IQR)* | 5.0  (5.0-7.0) | 6.5  (6.0-7.5) | z=-1.27  r=0.45  p=0.203 |
| Did you like the taste? (1=not at all, 10=extremely), median (IQR) | 4.0  (2.3-7.8) | 5.3  (4.0-6.0) | z=-0.28  r=0.1  p=0.778 |
| Was it pleasant to use? (1=not at all, 10=extremely), median (IQR) | 5.5  (3.3-7.5) | 5.8  (4.6-7.4) | z=-0.68  r=0.24  p=0.496 |
| How likely would you be to recommend it to friends? (1=not at all, 10=extremely), median (IQR) | 3.0  (1.3-5.0) | 7.0  (5.0-8.4) | z=-2.32  r=0.81  p=0.021 |

**N = 7 as one participant had missing data*

**Figure A: Urges to smoke after using IQOS, own brand cigarette and Juul (N=22)**
